# ‘Making a connection’: a qualitative study of experiences from a cancer telerehabilitation program

**DOI:** 10.1007/s00520-024-08803-w

**Published:** 2024-09-05

**Authors:** Amy M. Dennett, Nora Shields, Christian Barton, Allison Ezzat, Germaine Tan B. Physiother, Nicholas F. Taylor, Katherine E. Harding, Casey L. Peiris

**Affiliations:** 1 School of Allied Health, Human Services and Sport, La Trobe University and Allied Health Clinical Research Office, Eastern Health, Level 2, 5 Arnold St, Box Hill, VIC, Australia; 2 School of Psychology and Public Health, Olga Tennison Autism Research Centre, La Trobe University, Melbourne, VIC Australia; 3 School of Allied Health, Human Services and Sport, La Trobe University, Melbourne, VIC Australia; 4 Department of Physical Therapy, University of British Columbia, Vancouver, Canada; 5 Allied Health Clinical Research Office, Eastern Health, Box Hill, Victoria, Australia; 6 Allied Health Department, The Royal Melbourne Hospital and School of Allied Health, Human Services and Sport, La Trobe University, Melbourne, VIC Australia

**Keywords:** Telehealth, Telerehabilitation, Rehabilitation, Exercise, Implementation

## Abstract

**Purpose:**

Specialised group-based exercise rehabilitation is beneficial for cancer survivors but access to these services is limited. Telerehabilitation provides an opportunity to expand reach, but we do not know about the experiences of those who participate in this way. This study explored participant experiences of an exercise-based telerehabilitation program for people with cancer.

**Method:**

A qualitative study using semi-structured interviews was completed. Twenty-two cancer survivors were purposively sampled from the experimental group of a randomised controlled trial evaluating exercise-based cancer telerehabilitation delivered in groups using synchronous videoconferencing. Interviews were audio-recorded and transcribed verbatim. Data were coded independently by two reviewers and analysed inductively by thematic analysis.

**Results:**

‘A feeling of connection’ was the overarching theme. Participants perceived they connected with the health service, expert health professionals, and peers through participating in the telerehabilitation program. These connections provided a personalised rehabilitation experience and improved perceptions of physical and emotional well-being. Two subthemes suggested connection was facilitated by (1) the acceptability of telerehabilitation and (2) enhanced accountability to exercise. Participants felt disconnected when they were unable to participate in the program due to cancer treatment and side effects (e.g. fatigue), feeling unwell, and co-morbidities.

**Conclusion:**

We identified that telerehabilitation facilitated connections that enhanced the reach of exercise to cancer survivors. Our findings support using telerehabilitation to deliver specialised group-based exercise programs alongside more traditional models of care to increase participation in exercise among people with cancer.

**Supplementary Information:**

The online version contains supplementary material available at 10.1007/s00520-024-08803-w.

## Introduction

Clinical practice guidelines recommend the integration of exercise-based rehabilitation into cancer care [1–3]. The benefits of exercise include mitigating adverse treatment effects and improving physical function and quality of life [1]. Habitual exercise reduces the risk of cancer recurrence, mortality, and of developing chronic co-morbidities [4, 5]. Supervised exercise typically provided within structured rehabilitation programs facilitates the greatest benefit. Supervision improves adherence and participant outcomes compared to unsupervised programs [6]. Despite its benefits, translation of this evidence into clinical practice is poor, with suitable cancer rehabilitation programs available for just 1 in 200 people [7, 8].

Engaging cancer survivors in exercise-based rehabilitation is challenging. Attendance at rehabilitation programs is often impeded by treatment side effects [9, 10] and competing medical demands that leave people feeling overwhelmed [11]. Logistical challenges such as navigating complex referral pathways, cost, parking, and location further compound these issues, restricting access and limiting the effectiveness of exercise-based rehabilitation [10–12]. Convenience is a key facilitator to participation [11].

Providing exercise-based rehabilitation via telehealth (hereafter referred to as telerehabilitation) may overcome barriers to in-person rehabilitation. Technologies such as videoconferencing, wearable devices, and mobile applications can be used to replicate key elements of in-person cancer rehabilitation programs such as exercise demonstration, instruction, monitoring, and information provision. The safety, feasibility, and efficacy of telerehabilitation for individual cancer rehabilitation is well established [13–18]. However, exercise-based rehabilitation is commonly provided in group settings which can be an efficient way to deliver exercise and provide peer support, but might be challenging using telehealth.

Two preliminary studies indicate group-based cancer exercise programs are convenient but participants struggle to develop social connection [19, 20]. These studies were delivered early in the COVID-19 pandemic in community settings with participants who had been offered no choice in the transition from in-person exercise to telerehabilitation. No studies have explored the experiences of cancer survivors who choose to participate in group exercise-based telerehabilitation from earlier in their cancer journey. Establishing the acceptability and practicality of telerehabilitation from the patient perspective may help to facilitate the implementation of more flexible delivery of exercise programs for people with cancer, enhancing access to care. Therefore, the aim of this study was to conduct an in-depth exploration of the experience of participants of group exercise-based cancer telerehabilitation.

## Method

### Research design, theoretical framework, and setting

A qualitative study using interpretive description [21] was conducted to explore participants’ perspectives of cancer telerehabilitation. This study was embedded within a clinical trial evaluating the effect of a group exercise-based cancer telerehabilitation program (TeleCaRe) [22]. The trial was completed at a large, publicly funded health service extending from metropolitan Melbourne to regional Victoria, Australia. Participants provided accounts of their experience through semi-structured interviews. Data were collected between April 2022 and August 2023. The study was reported consistent with the Consolidated Criterion for Reporting Qualitative Research Checklist (COREQ) [23] checklist and approved by hospital and university ethics committees (E21-012–74698). All participants provided written informed consent.

### Participants

Participants were recruited from the experimental group of the TeleCaRe clinical trial (ANZCTRN12621001417875) [22]. Key eligibility criteria for the trial included participants who had cancer of any stage or type and were receiving, or within 12 months of treatment completion. Details of the intervention protocol and broader trial inclusion criteria are reported elsewhere [22]. Participants were purposively sampled to include people of various ages, genders, cancer types, and stages. Recruitment occurred via telephone and continued until there was adequate information power and no new ideas were identified during the interviews [24]. Participants could choose to have their carers present as a co-participant during interviews if they wished.

### Intervention

The intervention was an 8-week, twice-weekly group exercise program performed via videoconference under the direction of a physiotherapist. The program was supported by a physical activity device (Fitbit) and online information portal and home exercise program. All participants received an initial physiotherapy consult in-person before commencing telerehabilitation to ensure the safety of exercise. The program had a rolling enrolment of up to 8 participants per group. The duration of the sessions was 60 min.

### Semi-structured interviews

Participants completed interviews, via phone or videoconference (approximately 30 min), within 1 month of completing telerehabilitation. Interviewers were members of the research team (CB/AE) not involved with the delivery or administration of the intervention and were PhD-qualified physiotherapists experienced in qualitative research. A flexible interview schedule ensured relevant topics were covered while allowing participants to discuss their experience in the preferred order (Table 1).

**Table 1:** Interview guide

Topic area	Sample questions
**Cancer telerehabilitation** Participants to talk about their experiences and perspectives on the cancer telerehabilitation program	*What was your overall impression of cancer telerehabilitation?* *What was best about participating in cancer telerehabilitation?* *What elements of the telerehabilitation program were most useful? (Online portal, Fitbits, online group exercise, home exercise program)* *What elements of the telerehabilitation program were least useful?* *How could we improve the delivery of cancer telerehabilitation?*
**Accessing cancer telerehabilitation** Participants to discuss how they found accessing the program, including expectations	*Why did you decide to participate in this trial? (Prompt: what encouragement from your treating team was provided?)* *What did you think about participating in an exercise program delivered online before entering the trial? How did your attitude change as the program progressed? (Prompts: what telehealth for other appointments have you accessed before? What did you think?)* *How much effort did it take to organise everything you needed for each session? (Prompts: was there space, equipment, technology problems)* *What challenges did you experience with cancer telerehabilitation? How did you overcome these or what would help in the future to overcome these?*
**Group exercise via telerehabilitation** Participants to discuss their feelings about the type of therapy they received	*How ready did you feel to participate in a group exercise class online?* *How did participating in an online group exercise class affect your ability to be physically active? (For example, motivation, confidence, physical changes)* *How did you find the exercises provided in class? (Prompt: were they the right level for you? Was it flexible/adjusted over time and as needed?)* *How did participating in group exercise class *via* video affect therapist/patient interactions?* *Tell me about how you interacted with other patients participating in the online group exercise class. (Prompts: what (if any) support did you provide each other?)* *How did this program compare to other exercise experiences you have had? (Prompt: what advantages/disadvantages were there to delivering exercise online?) Would you recommend telerehabilitation to other people? If so, why? And if not, why not?* *How likely will you make or sustain changes to your exercise levels now the online exercise program is completed?*

### Data analysis

Interviews were audio-recorded and transcribed verbatim. Transcripts were de-identified and assigned an identification code for anonymity. Transcripts were read line by line, independently by two researchers (AD/CP), and open-coded (i.e. the codes were identified from the data) using NVivo (QSR International Version 12) software. Experienced PhD-qualified physiotherapy researchers (AD/CP/NS) completed thematic analysis by discussing codes during multiple workshops until consensus was reached on themes. Transcripts were re-read to selectively search for further data related to the identified themes and ensure no aspects had been overlooked. The principal researcher (AD) is employed by the health service where the study was conducted and has clinical experience in cancer rehabilitation while CP and NS are employed by an affiliated university and are experienced in qualitative research.

### Trustworthiness

Transcripts were sent to participants (member checking) to verify they were an accurate reflection of participants’ experiences [25]. Credibility and confirmability were established by having two researchers (AD, CP) independently code and reflect upon the data, enabling a thorough understanding of themes. Detailed descriptions of participants and setting enhanced dependability and enabled transferability [25].

## Results

Twenty-two participants were approached for interview and all agreed to participate. One carer also contributed to an interview with the participant. Participants were on average 64 years old (range 37 to 84) and most (*n* = 17) were receiving chemotherapy during telerehabilitation (Table 2). Participants attended an average of 11 out of 16 sessions (SD 4). The mean interview duration was 22 min (SD 7). During member checking, two participants returned their transcripts with minor grammatical amendments.

**Table 2:** Participant demographics

Gender – female, *n (%)*	13 (59)
Age (mean, SD)	64 (16)
Ethnicity, *n (%)*	
Caucasian	19 (86)
Asian	3 (14)
Diagnosis, *n (%)*	
Breast	9 (41)
Prostate	3 (14)
AML	2 (9)
Lymphoma	2 (9)
Other haematological malignancy	4 (18)
Other solid malignancy	2 (9)
Disease stage *n (%)*	
Early (0–III)	10 (45)
Advanced (IV)	4 (18)
Unknown	8 (36)
Receiving chemotherapy treatment	17 (77)
Total, *n*	22

### Main theme: *cancer* telerehabilitation facilitated connection

Participation in a group, exercise-based telerehabilitation program was described as an overwhelmingly positive experience that facilitated important, unexpected connections with the health service, rehabilitation experts, and peers.

“I felt like they [the physios] were actually making a connection and I didn’t expect that. I thought that it would just be a big group and I would just be one of a number.” (Participant S, 52 years old, breast cancer).

These connections resulted in improved self-reported patient outcomes including reduced fatigue, better functional capacity, and feelings of emotional well-being.

“I could feel myself getting stronger…and therefore applied myself outside the class to doing more and different things…as an example by the end of the course… I could do the lot [mowing the lawn].” (Participant N, 69 years old, amyloidosis).

Telerehabilitation appeared to connect with people who would not ordinarily access exercise-rehabilitation services including participants who had not previously participated in exercise.

“It got me doing exercise. Because historically, I hate exercise…” (Participant B, 80 years old, chronic myeloid leukaemia).

Once participants connected with telerehabilitation, many reported they overcame initial scepticism and enjoyed participating in exercise online. The importance of connection was reinforced by reports of negative experiences when participants became disconnected from the program (and associated benefits), due to treatment-related disruptions, other co-morbidities, and feeling unwell.

“I can’t really say I feel as though I benefitted [from telerehabilitation]…I missed a couple because of not being well enough and a couple of times because I had to go to hospital for the chemo…” (Participant J, 76 years old, chronic lymphocytic leukaemia).

Some participants found the shared experience of exercising with other people with cancer as helpful and reassuring.

“The experience of the other lovely people in the program, inspirational, because they clearly had all their own individual challenges. But they were so enthusiastic…and you catch that.” (Female carer of Participant D, 85 years old, prostate cancer).

However, many participants found building connection to other participants over video was challenging, but still felt able to make a connection to the physiotherapists who led the program. For many, connection with therapists and limited peer interaction via videoconference was sufficient.

“I felt a personal connection but not to anyone else in the class, only to them [the physios]... I didn’t actually feel any interaction or engagement [with other participants] but I didn’t want to or need to either. (Participant S, 52 years old, breast cancer)”

Participants valued the expertise provided by therapists, including their clinical and interpersonal skills. They described their ability to create an enjoyable and fun environment while providing clear and succinct exercise instruction and demonstration. This enabled participants to learn new knowledge and exercises to integrate into their recovery. Participants were particularly impressed with the ability of therapists to personalise exercise in a group, online setting by modifying exercise depending on their needs.

“They were very good at explaining, they gave you the alternatives…they took it at a nice pace…” (Participant H, 75 years old, lymphoma).

The initial face-to-face assessment further facilitated connection, which participants perceived to be as important for receiving information, education, and reassurance. Some perceived an in-person class would aid the tailoring of exercise and better facilitate social interaction compared to online and expressed a preference for such an option to be available. Nevertheless, participants weighed this desire for social interaction against the convenience of telerehabilitation and frequently reported they would participate in telerehabilitation again.

“I was very early on in my chemotherapy treatment. So I was also looking for a little bit of connection with people on my journey… you would be able to bond a little bit more in person than on a zoom call.” (Participant P, 44 years old, breast cancer).

### Subthemes

Two drivers of the connection were reflected in the subthemes: *(1) telerehabilitation was acceptable; (2) telerehabilitation enhanced accountability to exercise.*

#### Telerehabilitation was acceptable

The telerehabilitation program was acceptable to participants for a variety of reasons including its convenience to access, flexibility, and connection to therapists. Participants described the value of not having to travel and find parking which, for some participants, would have prevented them from accessing an in-person program. Participants of working age enjoyed the flexibility of fitting the program around work and family commitments, although some found the fixed times of the group a limitation. Many participants reported fatigue as a significant issue and so energy conservation was a primary advantage. Convenience was also helpful in aiding exercise participation despite feeling unwell as there was no need to travel.

“I wasn’t feeling well and having to log in on a laptop and doing the Zoom, at home, that was so easy. I didn’t need to drive anywhere; I could do it in my lounge room.” (Participant P, 44 years old, breast cancer).

Support from members of the cancer medical treatment team was a clear driver for some patients in enhancing the acceptability of the program and promoting engagement.

“I know what I have to do to be active and the messaging from the oncologist was really strong about that.” (Participant S, 52 years old, breast cancer).

Participants described a fear of contracting COVID-19 or other illnesses due to their compromised immune system and, therefore, preferred connecting online as opposed to in-person.

“with COVID I’m so paranoid… there’s no way I would go into a gym at the moment…” (Participant C, 40 years old, breast cancer).

Ease of online participation also influenced acceptability. While many participants were familiar with the videoconferencing software used, even those who were not rarely reported issues logging in to the class. The main technological issues described were weak internet signals and audio problems related to music. These issues were described as easy to resolve and participants appreciated the technical support provided by therapists. Limited preparation requirements including minimal need for equipment and space were also benefits.

“I just have my resistance band and my bottle of water… It was pretty easy, and you didn’t need that much space.” (Participant V, 39 years old, breast cancer).

Some participants liked monitoring their heart rate for safety purposes. The Fitbit provided was perceived as more difficult than other trial elements to use. Some participants described challenges reading and interacting with the small interface and charging the device. However, participants who owned their own smartwatch often reverted to using this for monitoring instead.

“It was a great reminder constantly through the week that you need to keep your exercise up and you can check your steps, your heart rate.” (Participant M, 60 years old, metastatic breast cancer).

Despite therapists being able to effectively modify exercises to suit participant’s ability, some perceived acceptability would be enhanced by offering a higher and lower-level group to cater for the broad range of abilities. Participants were also provided access to an online portal and virtual home exercise program app but reported not frequently using these resources.

#### Telerehabilitation enhanced accountability to exercise

Participants reported telerehabilitation positively influenced their physical activity behaviours. Classes held them accountable, with participants describing the program as an impetus to initiate or to return to exercise. They said classes gave them the time and discipline to exercise, resulting in establishing a routine. Regular class times and repetition created consistency participants felt they could adhere to. They also felt accountable to not letting down their therapist and peers.

“[The program] was just a really good routine…I could schedule in the time and there was an expectation and commitment to do it.” (Participant F, 42 years old, colorectal cancer).

Many participants expressed a desire to continue exercising beyond the program. Some acted on this positive intent by adding new exercises to their existing routines, increasing walking outside of classes, and exploring options for other exercise programs. A few participants purchased a smartwatch so they could continue to monitor their physical activity after the program. Others, particularly those who were older and still receiving cancer treatment, said they wanted to continue the supervised program and said they found discharge, or disconnecting, difficult as they enjoyed the new routine and accountability.

‘I really enjoyed it. I just didn’t want it to finish.’ (Participant L, 72 years old, breast cancer).

### Synthesis of findings

**Fig. 1:**
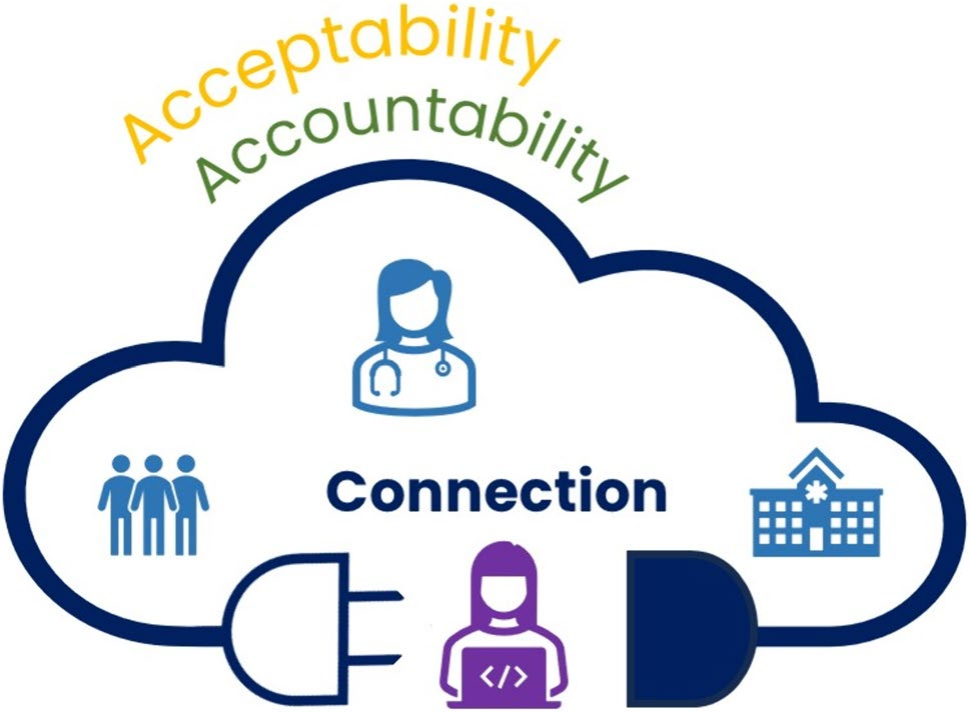
Interpretive synthesis of the connection of participants to the health service, therapists, and peers as facilitated by acceptability and accountability provided by telerehabilitation

An interpretive synthesis describes how the key drivers of acceptability and accountability of telerehabilitation facilitated connection (Fig. 1).

Telerehabilitation was an effective and acceptable way to facilitate connections between participants with cancer and health professionals, the health service, and others with shared experiences. Convenience was critical to its success and enabled participants to connect to the program even when traditional barriers to exercise such as fatigue, work, and family commitments were present. The interpersonal skills of the therapists were essential to the program’s acceptability, built personal connections with and between participants, aided adherence, and instilled a sense of trust, confidence, and hope.

The group structure also created accountability within and between participants. Regular class times, exercise familiarity, skilled therapists who created varied and appropriately modified exercises, and, for some, use of smartwatches supported this. Other elements such as the unsupervised home exercise program and online portal did not appear to play a role in making connections. Participants who were frequently unwell found it more difficult to build connections described by others as they could not attend regularly.

## Discussion

This qualitative study found participation in group exercise-based cancer telerehabilitation facilitated connections that improved access to this core component of cancer care. Key drivers of connection were acceptability and accountability, which was facilitated by skilled rehabilitation experts. Barriers to connection are often related to episodes of being unwell, which is also common in face-to-face programs [10]. Limited peer interaction online compared with equivalent in-person programs reduced opportunities for connection for some participants. This study adds to previous telerehabilitation literature by explaining the benefits of telerehabilitation beyond convenience, including connection and support.

The potential for telerehabilitation to connect cancer survivors has broad implications for improving access to exercise services. As with previous studies, convenience was a key factor in the decision to participate in telerehabilitation [11, 26] and telerehabilitation was able to reach people who may not participate in standard programs due to distance, mobility issues, or lack of enthusiasm. Missed sessions due to ill health were the main sources of disconnection but this issue is not unique to telerehabilitation. Telerehabilitation appeared more resilient to the issue of non-adherence than in-person rehabilitation as participants could still attend and connect even if feeling unwell. Adequate support to develop and promote telerehabilitation programs to connect patients is critical given the lack of exercise services to refer cancer survivors to is a major barrier to access [7, 12].

A key finding was that participants felt connected, safe, and able to benefit from exercise despite the remote nature of the interaction. Therapist interpersonal skills and expertise are described as critical elements of cancer rehabilitation [27]. This expertise ensured participants felt safe to exercise at home and modify their program as needed. Participants described similar benefits from telerehabilitation as those reported during in-person rehabilitation such as returning to meaningful activity [27]. This aligns with studies in other populations (such as pulmonary conditions) using novel group exercise delivery demonstrating telerehabilitation is not inferior to in-person rehabilitation [28, 29]. Our findings suggest cancer telerehabilitation can be provided with similar fidelity to in-person rehabilitation when staff are adequately trained, despite the inability to provide ‘hands-on’ support and having less equipment.

Despite the opportunities for connections offered by videoconferencing, some participants would have valued more social interaction with their peers. This issue is commonly cited in telehealth literature despite efforts to facilitate online interaction [20]. The inability to see some of the other participants, less time for incidental socialisation, and being limited to one circle of attention (i.e. talking to all participants) make it difficult for participants to engage with others one-on-one. It should be acknowledged both in-person and online exercise programs have advantages and disadvantages and online exercise may never be able to achieve the full social experience of in-person exercise. Therefore, when deciding on the mode of delivery for cancer telerehabilitation programs, it is important to consider patient preference and their need for social interaction. Ideally, offering both alternatives gives people the opportunity to choose options that best meet their needs. However, if the option of in-person exercise is not available, online groups are likely to be an effective alternative with attention to putting measures in place to facilitate group cohesion [30].

A positive outcome of the connection created by telerehabilitation was that it facilitated accountability and a commitment to ongoing exercise. In-person exercise programs may also facilitate accountability to exercise if combined with behavioural counselling like motivational interviewing [31]. However, transitioning exercise participation from rehabilitation to independent exercise in the community is often described as challenging [32, 33] as facilitators of behaviour change (e.g. social support, equipment, monitoring) are removed when rehabilitation is complete [34]. As telerehabilitation takes place in the participants’ home environment, it may offer an advantage over centre-based programs in enhancing habit formation, with less reliance on external motivation and greater facilitation of automaticity [35]. Therefore, exercise telerehabilitation may have lasting effects on physical activity participation that need to be investigated in future research.

### Strengths and limitations

This study was reported using the COREQ checklist and strategies were employed to ensure rigour and trustworthiness, including independent coding by two reviewers and member checking. We believe we achieved adequate information power as no new ideas were evident in the final eight transcripts [36]. The study was conducted within a large health service setting using purposive sampling to identify people from a broad age range and cancer experience that enhances generalisability to similar health settings.

Given participants were recruited from a clinical trial, participants may have been motivated to exercise resulting in selection bias which may overestimate our findings supporting the benefits of telerehabilitation. However, our data showed many participants were novice exercisers. Data were also limited to participants from one health service located in a public system with no cost to participants. Experiences may differ in other health systems or rural and remote areas where there is lower digital literacy and access to reliable internet.

## Conclusion

Exercise-based cancer telerehabilitation facilitated connection between participants with cancer and the health service, rehabilitation experts, and their peers. Telerehabilitation was a positive experience where connection was facilitated through acceptability and accountability. This study suggests telehealth may be an effective addition to traditional models of care delivery to improve access to exercise-based cancer telerehabilitation.

## Supplementary Information

Below is the link to the electronic supplementary material.

Supplementary file1 (DOCX 30 KB)

## Data Availability

All data supporting the findings of this study are available within the paper and its Supplementary Information (Supplementary file 1).
