# Supplementary material for: ‘Making a connection’: a qualitative study of experiences from a cancer telerehabilitation program
Source: Support Care Cancer. 2024 Sep 5;32(10):636. doi: 10.1007/s00520-024-08803-w (PMC11377521; doi:10.1007/s00520-024-08803-w)
Supplement: Supplementary file 1 — Supplementary file1 (DOCX 30 KB) [file 520_2024_8803_MOESM1_ESM.docx]

**‘I felt like they were making a connection’: A qualitative study of experiences from a cancer telerehabilitation program**

Amy M Dennett School of Allied Health, Human Services and Sport, La Trobe University and Allied Health Clinical Research Office, Eastern Health, Victoria, Australia [amy.dennett@easternhealth.org.au](mailto:amy.dennett@easternhealth.org.au), Nora Shields, Christian Barton, Allison Ezzat, Germaine Tan, Nicholas F Taylor, Katherine E Harding, Casey L Peiris

Supplementary file 1: Selected quotes

| *Main Theme* | |
| --- | --- |
| **Telerehabilitation facilitates connection** | - *“I do think it did me good because I might have started off the morning sluggish. You know ‘I can't be bothered’ …You just felt better when after you've done it…more able to cope with the rest of the physical stuff through the day… I felt good about myself and the fact that, I did have the energy to keep on going*.” (Participant A, 84 year old, myelodysplasia) - “*I could feel myself getting stronger…and therefore applied myself outside the class to doing more and different things…as an example by the end of the course… I could do the lot [mowing the lawn]*.” (Participant N, 69 year old, amyloidosis) - *“It helped me get a little bit out of myself. I just kept doing the systems, concentrating on the exercises…”* (Participant M, 60 year old, metastatic breast cancer) - “*It really helped me shift into a state of hope*” (Participant S, 52 year old, breast cancer) - *“Once I realised, I'm doing l these little squat things…it was actually quite rewarding given, how much lack of control of the rest of my body…mentally it was actually really cool to be able to go hey, I was getting something out of this!”* (Participant V, 39 year old, breast cancer) - *“I think the fact that it got me going again and it got me doing exercise. Because I used to be quite - historically, I hate exercise… if you're online, and you're having a problem. I think you feel less like you’re wasting people’s time.”* (Participant B, 80 year old, chronic myeloid leukaemia) - *“I just have to do this for wellbeing, I know what I have to do to be active. And the messaging from the oncologist was really strong about that. So this provided the opportunity to back it up*.” (Participant S, 52 year old, breast cancer) - *“[The physios] made it really easy to connect to the program. Coming, dropping off the tracker and the theraband, the initial assessment, explaining everything and then the post assessment and explaining the next steps. I think it was really structured well*.” (Participant P, 44 year old, breast cancer) - *“[The physio] is amazing. She is just so good at her job. And I always felt really supported… she was so kind to include my children. It's like, it just made it easy for me to join in.”* (Participant C, 40 year old, breast cancer) - “*I felt like they were actually making connection. And I didn’t expect that, I thought that it would just be a big group. And I would just be one of a number…absolutely credit to those physios. They did a great job of connecting with people.”* (Participant S, 52 year old, breast cancer) - “*you felt acknowledged without being called out… I did feel safe, like, someone was keeping an eye on me, but not calling me out…and they were very kind.”* (Participant V, 39 year old, breast cancer) - *“I think is probably a bit easy for me. But if I look at the balance of where the line needs to be, for most of the group members, it was it was pretty good.”* (Participant F, 42 year old, colorectal cancer) - “*I was very early on in my chemotherapy treatment. So I was also looking for a little bit of connection with people on my journey… you would be able to bond a little bit more in person on a zoom call, it's harder to build those connections and not everyone's looking for that*.” (Participant P, 44 year old, breast cancer) - “*I felt a personal connection but not to anyone else in the class, only to them [the physios]...familiar names came up and I thought that's nice...I didn’t actually feel any interaction or engagement but I didn't want to or need to either.”* (Participant S, 52 year old, breast cancer) - “*the experience of the other lovely people in the program, inspirational, because they clearly had all their own individual challenges. But they were so enthusiastic…and you catch that*.” (Female carer of Participant D, 85 year old, prostate cancer) - “*my understanding was everyone there had had chemo. So knowing that and then meant that I didn't feel bad if I wasn't feeling up to it, or I didn't feel guilty or pressured or whatever.”* (Participant I, 37 year old, breast cancer) - “*I didn't gain as much benefit as I could have because of the fact I just wasn't I just wasn't strong enough during the during the course of the of the program to do it properly*.” (participant H, 75 year old, lymphoma) - “*They were very good at explaining, they gave you the alternatives… they took it at a nice pace… emphasising all the time not to kill yourself, type of thing!”* (Participant H, 75 year old, lymphoma) |
| *Subthemes* | |
| **Telerehabilitation is acceptable and convenient** | - “*It [travelling to and from in-person class] disrupts my day too much. You’ve wasted say about 3 hours, 4 hours by the time you get out you drive down to wherever you have to go and do your exercises and you come back and can’t be bothered because half your day is gone but that was a great advantage to me and living out here…having it online … because it didn’t really stuff the day up totally.”* (Participant A, 84 year old, myelodysplasia) - *“To have to go anywhere would be the deal breaker for me. And I think, I think in cancer treatment, you just haven't got any energy to do that. And that's why I love the Zoom classes, because I wouldn't have had to drive somewhere to find parking. Your life is so full of friggin appointments anyway.”* (Participant S, 52 year old, breast cancer) - *“It wasn't the same mundane exercises all the time. We had a few different ones brought in every now and again. And that made it much better*.” (Participant A, 84 year old, myelodysplasia) - *“Online option is just perfect… you're in and out of hospital enough… I feel like that would just be one extra thing that you probably wouldn't feel like doing. Like I personally wouldn't want another trip to the hospital do an exercise class.”* (Participant C, 40 year old, breast cancer) - “*If it was down the road I would prefer in person. But if it was a half hour drive or more? No.”* (Participant E, 72 year old, acute myeloid leukaemia) - “*The fact that you don't have to travel anywhere and just added the extra fatigue into that whole thing for me was, was really important. So, I felt like I could give my energy into the session rather than kind of tiring myself out getting there and getting home.”* (Participant F, 42 year old, colorectal cancer) - *“I still prefer the Zoom platform actually. Normally, I would say I prefer being in person. Being back at work, I wouldn't have had the time to drive. I would have missed too much work [in person].”* (Participant I, 37 year old, breast cancer) - “*This was a really good time for me. And I will say this when I first started, I was deliberating as to whether it was going to meet my expectations, but it certainly did*.” (Participant N, 69 year old, amyloidosis) - “*I wasn't feeling well, and having to log in on a laptop and doing the Zoom, at home, that was so easy. I didn't need to drive anywhere, I could do it in my lounge room. So that was amazing for where I was at.*” (Participant P, 44 year old, breast cancer) - *“it was flexible ... I could actually do it during my working hours*.” (Participant S, 52 year old, breast cancer) - “*Once [physio] set me up with Zoom because I had never done a Zoom meeting... I had no problem.”* (Participant L, 72 year old, breast cancer) - “*It was professionally ran. I really enjoyed it. I just didn’t want it to finish*.” (Participant L, 72 year old, breast cancer) - “*I just have my resistance band and my bottle of water and I had either my mum or my dad then they came across so that that they were there if I needed help, help wrangle my dog. it was pretty easy and you didn't need that much space, it was pretty adaptable.”* (Participant V, 39 year old, breast cancer) - *“The Fitbit I had no idea how to use but I've got a Garmin that I use. As for user friendly, the Fitbits they were hard and I’m pretty tech savvy*.” (Participant V, 39 year old, breast cancer) - *“I’m not very good with the computer. A couple of times I just did something wrong to log on. But you know, we sorted that out.”* (Participant O, 70 year old, prostate cancer) |
| **Telerehabilitation enhances accountability to exercise** | - *“You have to do the hour... It wasn't a chore and the further it went on, the better it got.”* (Participant A, 84 year old, myelodysplasia) - *“I'm still wearing the Fitbit, which, which I think has has been a useful check…and trying to make sure that I try and maintain my activity level a bit…that will give me a bit of a kick along.”* (Participant B, 80 year old, chronic myeloid leukaemia) - “*Having the discipline and the time to do regular exercise was just a really good routine…I could schedule in the time and there was an expectation and kind of a commitment to do it*.” (Participant F, 42 year old, colorectal cancer) - “*That organised hour twice a week... put a little bit of structure and I knew I have to do it. So sometimes, I wasn't feeling even that well, but I pushed myself and I did it.”* (Participant G, 72 year old, breast cancer) - “*The home exercises would be great if I did them. But that’s why I needed the accountability because I know I’m rubbish at doing things.”* (Participant I, 37 year old, breast cancer) - “*I think the main motivator was because my job's quite physical. So I wanted to get, you know, moving again, quickly, so that I can do the things I need to do at work. And also because I've got young kids who I still need to sometimes carry and missed*”. (Participant I, 37 year old, breast cancer) - “*I'm a bit lazy. So I really look forward to the Zoom meetings on the Tuesday and Thursdays where I did the exercises.”* (Participant L, 72 year old, breast cancer) - “*It's consistency. But then I was hoping that I could do like twice a week. But then because they only do it on Tuesday, Thursday, the Thursday between my treatment days, so it's very hard to make it. But then it's good to still have that.”* (Participant Q, 39 year old, breast cancer) - *“That just encouraged me to move because … I wasn't feeling well enough … so I've kind of felt, okay, I've got a session, it's only for an hour, I'll just mentally prepare and do it. And that got me off the couch or out of bed or whatever. And then I always felt better once I'd had done my sessions*.” (Participant P, 44 year old, breast cancer) - *“It was a very loose target. But I was just conscious of beating like the day before or whatever my highest steps were for the week.”* (Participant P, 44 year old, breast cancer) - “*The Fitbit I thought was a terrific idea I quite warmed to the Fitbit… it was a great reminder, constantly through the week that you need to keep your exercise up and you can check your steps, your heart rate.”* (Participant M, 60 year old, metastatic breast cancer) - “*Fitbit was good because I was having a bit of tachycardia with the drugs. And it was good because it's giving me my resting heart rate … over Christmas, they're on special for $70. Which I'm going to use just to monitor the heart rate right and just steps*.” (Participant T, 73 year old, multiple myeloma) |
